# Selecting EHR-driven recruitment strategies: An evidence-based decision guide

**DOI:** 10.1017/cts.2022.439

**Published:** 2022-08-08

**Authors:** Randall W. Grout, Dan Hood, Sarah J. Nelson, Paul A. Harris, Peter J. Embí

**Affiliations:** 1 Indiana University School of Medicine, Indianapolis, IN, USA; 2 The Regenstrief Institute, Inc., Indianapolis, IN, USA; 3 Vanderbilt Institute for Clinical and Translational Research, Vanderbilt University Medical Center, Nashville, TN, USA; 4 Department of Biomedical Informatics, Vanderbilt University Medical Center, Nashville, TN, USA; 5 Department of Biostatistics, Vanderbilt University Medical Center, Nashville, TN, USA; 6 Department of Biomedical Engineering, Vanderbilt University, Nashville, TN, USA

**Keywords:** recruitment, EHR, patient portal

## Abstract

Participant recruitment for research is a persistent bottleneck that can be improved by leveraging electronic health records (EHRs). Despite emerging evidence for various EHR-driven approaches, guidance for those attempting to select and use such approaches is limited. The national Recruitment Innovation Center established the EHR Recruitment Consult Resource (ERCR) service line to support multisite studies through implementation of EHR-driven recruitment strategies. As the ERCR, we evolved a guide through 17 consultations over 3 years with multisite studies recruiting in diverse biomedical research domains. We assessed literature and engaged domain experts to identify five key EHR-driven recruitment strategies: direct to patient messages, candidate lists for mailings/calls, direct to research alerts, point of care alerts, and participant registries. Differentiating factors were grouped into factors of study population, study protocol and recruitment workflows, and recruitment site capabilities. The decision matrix indicates acceptable or preferred strategies based on the differentiating factors. Across the ERCR consultations, candidate lists for mailing or calls were most common, participant registries were least frequently recommended, and for some studies no EHR-driven recruitment was recommended. Comparative effectiveness research is needed to refine further evidence for these and potentially new strategies to come.

## Introduction

Suboptimal participant recruitment for clinical research is a barrier to advancing science and discovery [1,2]. The Trial Innovation Network [3], and specifically the Recruitment Innovation Center (RIC) within it [4], funded by the CTSA program of the NCATS, was tasked with developing and disseminating innovative methods to improve [4] the efficiency, effectiveness, inclusiveness, recruitment, retention, and successful completion of trials. Electronic Health Record (EHR)-driven recruitment approaches are increasingly being used [5,6]; more than half of randomized controlled trials since 2000 that involved the EHR used EHR-driven recruitment, including 82% of trials with a non-EHR intervention [7]. However, relatively little guidance exists to support use of one EHR-driven recruitment strategy over another, and the use of an inappropriate strategy can lead to poor enrollment rates and/or wasted resources. To address this need and gap in the literature, we aimed to develop an evidence-based approach to the selection and use of EHR-driven strategies for participant recruitment.

Both EHRs and clinical data repositories can help enhance research planning and recruitment through site identification, study candidate identification, predicting enrollment, and facilitating recruitment [5,8]. These tools can be leveraged to identify patients prior to an upcoming visit, deploy resources to invite patients in clinical settings, or expedite-directed communications (e.g., mailing and call lists). However, these methodologies have been limited because research is often not considered an essential part of the culture of routine practice, informatics-based tools must be seamlessly integrated into the clinical workflow to be successful, and considerable variation exists with respect to a site’s maturity of their technical infrastructure. Planning for EHR-driven recruitment strategies in multisite studies is challenging given the diversity of EHR vendors, recruitment support modules across sites, local data governance policies, as well as other site-specific factors. Perhaps the most important limitation is that research teams lack an adequate guide to determine which (if any) EHR or clinical data repository-based recruitment tool is appropriate to use for their study.

**Fig. 1.**
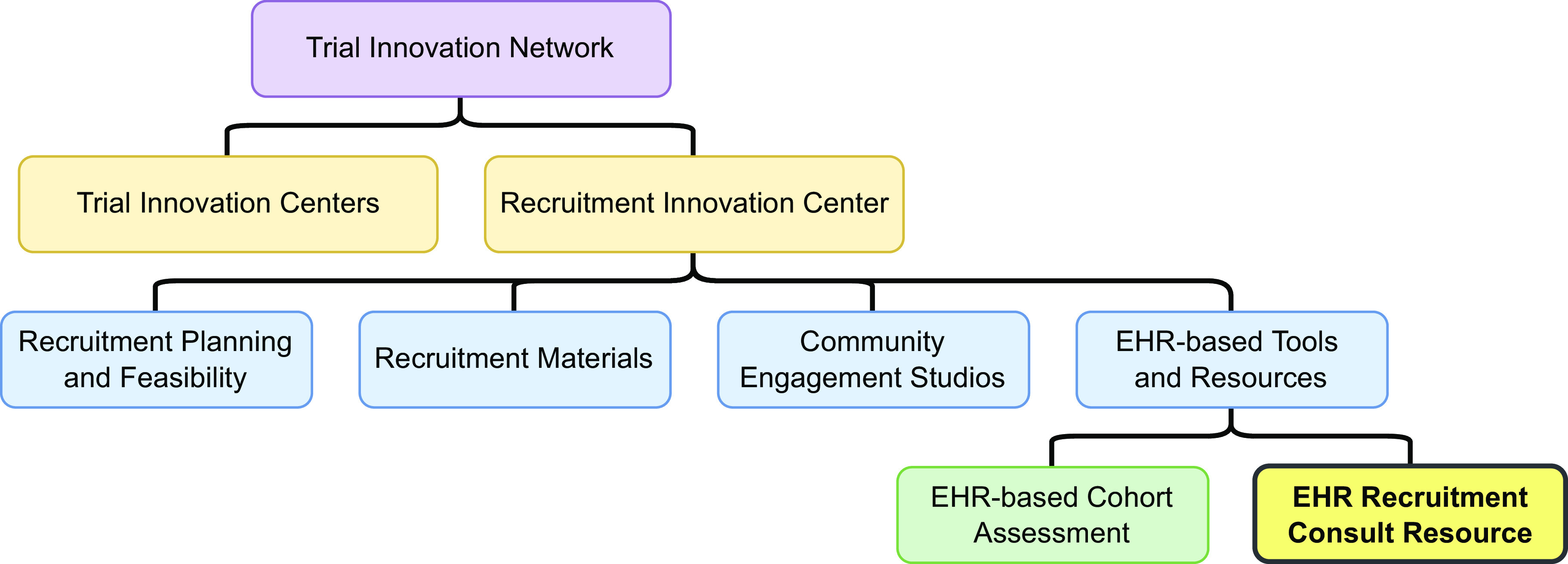
Organization chart of the electronic health record (EHR) recruitment consult resource within the recruitment innovation center.

To help address these limitations, the RIC developed a EHR Recruitment Consult Resource (ERCR) service line in 2017 to support trials requesting assistance through the Trial Innovation Network [3,4]. The ERCR works directly with clinical study teams and their recruitment sites to better understand their site-specific recruitment workflows, IT capabilities, and study protocol to provide recommendations to enhance recruitment efforts using EHR-based tools. Notably, the ERCR is part of a larger suite of services supporting trial recruitment, including the EHR-based Cohort Assessment service line for study site selection (see Fig. 1 for organization chart) [9]. The ERCR includes domain experts and project managers to support consultations. The ERCR developed a guide to support engagement and decision making with study teams to better understand their recruitment needs and tailor recommendations based on several criteria. We applied this guide and associated decision matrix to several RIC sponsored studies. Engagement with a diverse set of study teams allowed for continuous iteration of our approach to develop a more generalizable guide for future consultations. The guide should provide value to any medical center team supporting recruitment in single or multisite clinical studies and trials.

## Guide Development

### Landscape of EHR-driven Recruitment Strategies

Based upon a review of the literature related to EHR-driven recruitment and collection of firsthand feedback using some of the methodologies from our team’s content experts, we began by developing a list of EHR-driven recruitment approaches. Then, we conducted a series of open-ended group discussions with content experts from the Indiana University CTSA Research Network (ResNet) to elicit their experiences and internal heuristics for the selection and use of specific EHR-driven recruitment strategies. Individual approaches were categorized into one of five groups which are agnostic to a specific EHR platform or data warehouse but represent a range of EHR-driven approaches which could be leveraged by individual study sites.

### Decision Guide with Differentiating Factors

We searched for literature-derived considerations for the use of certain EHR-driven recruitment strategies. We also engaged with a subset of research assistants from ResNet who had an experience base of approximately 20 person-years working with teams of health services researchers, behavioral scientists, and medical providers to offer support in identification and recruitment of study participants. We created differentiating factors that included lists of questions intended to collect information specific to the study population, the study intervention, and the technical capabilities of the intended recruitment sites. Based on guidance from the literature and content experts on our team, we developed a decision guide which mapped responses to these questions with recommended use of one or more EHR-driven recruitment strategies. It was introduced in ERCR consultations to engage with study teams and collect information and requirements on EHR-driven recruitment strategies. The questions were answered via protocol review and direct communication with the study team, and they informed the ERCR recommendation to use one or more EHR-driven recruitment strategies, or in some cases, to use none.

### Iterative Approach to Incorporating Additional Intake Criteria

The recruitment tool questionnaire and decision guide were not considered static documents and were updated through continuous iteration as informed by engagement with study teams. With each consultation, documentation was updated to expand on the information collected to create a more representative guide.

### Peer Debriefing

In order to evaluate the validity of the information collected and guide established, we utilized a qualitative research tool known as peer debriefing [10]. Members of the RIC informatics development subgroup, who are familiar with EHR-based recruitment methodologies, evaluated the guide for accuracy and validity. We also engaged independent experts for input on the guide. Upon completion of peer debriefing, we identified points of agreement, confirmations, changes to the decision guide, and any questions raised. The latest guide was mutually agreed upon by this subgroup and is presented here.

## The Decision Guide

Our review of EHR-based recruitment strategies from prior literature and expert/peer discussions produced five approaches:

1. Direct to patient messages
2. Candidate lists for mailings/calls
3. Direct to researcher alerts
4. Point-of-care alerts
5. Participant registries

These were not exclusive with one another in each study; multiple approaches could be used.

Between 2017 and 2021, we completed 17 ERCR consultations. Table 1 shows the frequency of recruitment strategy recommendations. Participant registries were less frequently recommended, while other strategies were common. Notably, there were four instances where EHR-driven recruitment was not recommended due to lack of anticipated benefit for the trial.

**Table 1.** Electronic health record (EHR)-driven recruitment strategy recommendations across 17 EHR Recruitment Consult Resource consultations

Recruitment strategy	Number of recommendations*
Direct to Patient	5
Direct to Researcher	4
Point of Care	4
Candidate Lists	6
Participant Registries	2
None	4

* Some consultations produced multiple recommendations.

The EHR-driven recruitment decision guide served to 1) standardize ERCR consultation and walk study teams through a series of questions which were pertinent to recruitment planning; 2) introduce the study teams to various EHR-based recruitment strategies and capabilities; and 3) use data collected from 1 and 2 to propose recruitment strategies. The guide is presented as a matrix of recruitment strategies across differentiating factors in Table 2.

**Table 2.** Electronic health record (EHR)-driven recruitment strategy decision guide

	Direct to Patient (portal messaging)	Direct to Researcher Alert (DRA)	Point of Care Clinical Trial Alerts (CTA)	Candidate Lists for Mailings or Calls	EHR-based Patient Registries
**Factors of study population**					
Study disease/condition is rare	○	●	●	○	●
Study disease/condition is common	●			●	
Eligible patients frequently interact in healthcare setting	●	●	●	○	
Sensitivity or stigma associated with disease/condition	○	●	●		
Small recruitment pool per site	○	●	●	○	●
Large recruitment pool per site	●	○		●	
**Factors of study protocol and recruitment workflows**					
Inclusion/exclusion criteria are readily available in EHR	●	●	●	●	●
Study intervention or randomization is taking place in an inpatient setting		●	●		
Study intervention or randomization is taking place in an emergency care setting		○	●		
Study intervention is taking place in an ambulatory or virtual setting	●			●	○
Study intervention or randomization requires real time identification of patient		●	●		
Intervention or study requirements are easily understood by participant	●			●	
Study requires immediate implementation of methodology for immediate recruitment	○			●	
**Factors of recruitment site capabilities and policies**					
Site can query EHR or health system data warehouse	●	●	●	●	●
Site has limited capacity for development within EHR system	●			●	○
Site has immature EHR infrastructure				●	
Site has access to informatics or health information technology resources to support recruitment	●	●	●	○	●

○ Acceptable strategy.

● Preferred strategy.

### EHR-driven Recruitment Strategies

At their core, all EHR-driven recruitment strategies require a computable phenotype [9,11]. The phenotype may be general (“all patients ages 2–18 years”) or specific (“4-year-old females with an encounter diagnosis ICD-10-CM code F84.0 from clinic X in the past 30 days”) and should be well specified. The degree to which logic rules can be specified and combined with existing data to identify study candidates determines the efficiency of EHR-driven recruitment. These automated criteria substantially reduce the manual screening efforts. They may require validation and bias assessment [12,13].

### Direct to Patient Portal Messaging

Direct to patient portal messaging uses the existing patient portal clinical message feature, often used for provider-patient communication, sharing notes, and viewing lab results, to invite study candidates to participate. This approach facilitates recruitment in a flexible, cost-efficient approach for many different types of studies. It is well received by patients and may lead to faster, cheaper recruitment than traditional phone calls or letters [14]. It usually requires modest upfront technical development in order to integrate eligible participant lists with the EHR portal platform used at recruitment sites. The message recipient may also receive a mobile push notification or an email. Patient portals can be especially helpful to automate distribution of communications to eligible participants [15–17]. While studies which target small numbers of enrollees can benefit from this approach, a larger advantage is in studies which target medium to large pools of participants because of the automated approaches. Some patient portals allow patients to express interest in particular types of research studies or search for studies seeking enrollment. Though relatively small in scope now, future recruitment efforts may leverage this patient-motivated study matching, with caution for the potential biases or disparities related to this selected population [15].

Direct to patient portal messaging may not be suitable for studies which need to rapidly identify eligible participants in a clinical setting. Ideal protocols for this approach include interventions which may take place in an ambulatory or virtual setting, though in-clinic recruitment may perform better [18]. Researchers should consider the overall complexity of the intervention and subsequent study requirements. Studies which employ this approach must clearly and concisely convey the requirements of the study through asynchronous, written communication. Complex study interventions may be best communicated in person, rather than through a virtual platform. Recruitment sites may also have limitations on how EHR portals may be used for research [19]. Not all patients enroll in a patient portal, and not all enrollees actively use it. Finally, this approach does require internet access and may introduce other biases in the study population [15,20].

### Candidate Lists for Mailings or Calls

Query-derived patient lists for mailings or calls represent a flexible and well-known approach to recruiting eligible patients. Accurate patient lists require site-specific informatics resources to run the computable phenotype and transmit the patient list to research teams. However, this approach does not require direct development within the EHR technical infrastructure and generally has a smaller IT footprint when compared to other strategies.

Patients lists for external use are not suitable for studies which require rapid or real-time identification of patients in the clinical setting. Ideal candidates for this approach include interventions which may take place in an ambulatory or virtual setting, or asynchronous from clinical care. Additional consideration should be given to the overall complexity of the intervention and subsequent study requirements. Studies which employ this approach must communicate clearly and concisely to explain the requirements of the study, especially when using asynchronous recruitment contact. Institutions may vary in their research contact policy regarding which patients can be contacted and by whom.

### Direct to Researcher Alert

A direct to researcher alert to facilitate recruitment is a more targeted approach for recruitment which takes place in the clinical setting. These may take the form of push notifications or a refreshable task list [21]. If adopted, a recruitment site would need to invest upfront resources to ensure that queries necessary to identify eligible patients are embedded into workflows which can rapidly communicate patient lists to researchers or recruitment coordinators. This approach can leverage prebuilt querying infrastructure which exists in most commercially available EHR platforms or draw insight from queries written against a health system’s enterprise data warehouse. However, data warehouse queries may not provide real-time notifications due to data extraction delays. In either scenario, health IT teams, and/or local informatics groups should be engaged to support implementation.

This approach is ideal for recruitment which takes place in the inpatient setting, as direct to researcher alerts can allow for rapid identification of eligible participants. This strategy can support recruitment for common presentation of certain disease or morbidity but may be most advantageous for identification of rarer conditions which are less often seen in the healthcare system, and the researcher is on-call for a recruitment opportunity.

### Point of Care Alert

Point of care alerts represent a highly targeted approach for recruitment which takes place in the clinical setting. A point of care alert triggers a message to the health care provider, generally within a patient’s EHR, and conveys study information or the patient’s eligibility status for a given study. If adopted, this approach requires initial investment and allocation of IT resources as well as coordination with health system IT leadership. Further, clinical leadership must be involved to represent and align with the clinical workflow. Efforts to design and implement a point of care alert can vary depending on the complexity of the eligibility criteria and planned interactions.

Early studies of point-of-care clinical trial alerts demonstrated their effectiveness at increasing recruitment to studies for chronic conditions in ambulatory settings [22]. Though generally helpful for recruitment, point of care alerts may especially help immediate identification of a patient, including those which may be identified in an acute (ED or inpatient) setting [23]. Additionally, studies may benefit from introduction by the health care provider or leverage their endorsement are well suited for point of care alerts. Studies which have common eligibility criteria may want to avoid the point of care alert, because though clinical trial alerts are generally accepted [24], higher levels of identified patients can create “alert fatigue” among healthcare providers, causing the alert to be less effective [25].

### EHR-based Patient Registries

The development of a patient registry or use of an existing registry to facilitate recruitment can be an effective means of identifying patients which are representative of a certain population. Some registries, especially multisite registries, may be populated by extracting EHR data from patients with a specific disease or shared characteristic. Registries may also live within an EHR and be continuously updated. Studies which recruit patients with rare disease can benefit from the use of a participant registries targeting that same population.

A patient registry differs considerably when compared to the other EHR-driven strategies discussed. In registries, the data available for phenotyping patients may be limited to what has already been extracted from the EHR. Integrated registries may offer more flexibility by still connecting to other EHR data. Still, the availability of patient registries as predetermined cohorts, especially in rare conditions, is an important component in the overall research recruitment toolkit.

### Differentiating Factors

The decision guide focused on identifying differentiating factors that support the use of one EHR-based strategy over another. Depending on the study protocol, multiple approaches may be appropriate. Separate methods may be needed per site, and investigators should ensure adopted strategies will not interfere with trial analysis and outcomes. Patient, clinician, IRB, and researcher perspectives should all play a role in selecting and optimizing EHR-driven recruitment strategies [24,26,27].

### Factors of Study Population

A basic feasibility assessment [9] can often confirm whether a recruitment site has an adequate patient population to meet their recruitment goals. The rate at which an eligible patient population presents in the healthcare setting can be a strong differentiator between clinician directed point of care alerts, researcher directed alerts, other EHR based methods, or using non-EHR recruitment methods. Patients who infrequently use the health system may have outdated contact information returned for extracted lists or may not be active in the patient portal. Embedding a clinician alert within the EMR workflow may not be appropriate when the eligible population has a higher prevalence. The large technical resources necessary to implement such an approach coupled with a strong potential for alert fatigue can make this approach untenable for most recruitment sites. Similar consideration should be given for rare conditions, in which eligible patients may present to the healthcare setting at a lower prevalence. While alert fatigue may not be considered a concern, infrequent alerts with inadequate context may be dismissed.

Depending on the timeliness of the intervention, highly prevalent conditions can support the use of direct to patient messaging through an EMR portal, generation of subject lists for large-scale mailings/calls, or direct notifications to research team members. Recruitment of individuals with rare conditions might use existing patient registries, develop new registries, or direct notifications to research team members.

### Factors of Study Protocol and Recruitment Workflows

EHR-driven recruitment strategies should be aligned with the recruitment workflow. The study intervention’s setting and timing (after participant identification) can help define the most pragmatic approach and rule out unfeasible strategies. Protocols which target participants in the emergency department or inpatient setting require a more rapid screening process and timely access to computed eligibility criteria. In either setting, a strategy which employs a targeted point of care alert to the clinician or notification to the research team will be more effective. The time window of placing a participant into an intervention arm may also differentiate strategies as asynchronous communication may delay group assignment or misalign with randomization methods. Some recruitment strategies will query a health system’s data warehouse that has an inherent latency (e.g., 24 hour refresh cycle). Protocols which require identification of a participant in real time should consider the site-specific capabilities of querying the EHR system directly.

Alternatively, recruitment workflows in the outpatient or virtual setting may be more flexible to alternative EHR-driven strategies. These settings may support direct to patient messaging through health portals, general EHR data queries, or cohort identification for asynchronous participant contact through mail or phone, or targeted researcher alerts to contact participants in the outpatient setting.

### Factors of Recruitment Site Capabilities and Policies

Local recruitment site policies and capabilities may be the most stringent differentiating factor for considering EHR-driven recruitment strategies in support of multisite trials [6]. Selecting one or more strategies must be aligned with the resources available.

Research teams must rely on the data elements which comprise the clinical phenotype of the recruited population. Unstructured data can and should be used to support patient identification; however, natural language processing for recruitment requires advanced expertise and can be difficult to scale across multicentered studies [28]. Fortunately, previous work indicates that ∼75% of critical data necessary to determine eligibility is likely associated with structured data [11]. We identified that studies which rely on capture of social determinants of health or patient behaviors such as smoking may find difficulty relying on medical records. In addition, conditions with poorly defined computable phenotypes may find difficulty identifying the correct patients. Prior to committing to the use of an EHR-driven approach, research teams should evaluate their study eligibility criteria and perform initial feasibility assessments to ensure that necessary criteria are captured and well populated.

As large, multisite studies aim to leverage data-driven solutions to enhance recruitment, a standard single solution across multiple sites becomes more difficult. While the research community cannot expect health systems to adopt a standard data system or set of governance policies, the RIC ERCR has sought to shine a light on these variations during consultations with RIC-sponsored studies. When applicable, the RIC ERCR develops data collection instruments focused on study-specific requirements which study teams can disseminate as part of a site recruitment questionnaire or readiness assessment. The results support planning efforts and overall strategy for an EHR-driven recruitment plan. The process of collecting this information can ensure study teams are engaging with the necessary experts at their site who can ultimately support implementation. Regardless of the site or its technical maturity, all EHR-driven recruitment strategies require collaboration with local informatics professionals.

Access to EHR-driven recruitment capabilities is mediated by local policies and governance. Academic and healthcare institutions may have research policies directing the method, person, and frequency by which patients are contacted. For example, institutions vary in opt-out versus opt-in policies for research contact or data use [6]. These policies are developed while balancing institutional priorities of public trust, legal compliance, scientific advancement, and others [29]. Local research informatics professionals, such as a Chief Research Informatics Officer [30], can guide researchers in specific allowances or restrictions for EHR-driven recruitment.

### Limitations

The decision guide represents an initial approach and is meant to iterate and evolve as new strategies evolve. The current version is not intended to comprehensively inventory the site-specific maturity and technical infrastructure necessary to implement a given recruitment strategy. Nor do the strategies presented here represent all recruitment strategies; the current scope addresses methods that are most directly tied to the EHR. Blank cells do not mean the strategies will not work in the given circumstance. These strategies are highly generalized and may not be representative of all health systems across the USA or in other countries.

## Conclusions

EHR-driven approaches to participant recruitment have potential to improve recruitment rates, but not all approaches are appropriate to all studies or settings. To our knowledge, this is the first guide for EHR-driven recruitment approaches based upon best evidence (i.e., literature and expert opinion). We continue to evaluate this approach via studies utilizing RIC ERCR service line. The nascent evidence on EHR-driven recruitment will benefit from comparative effectiveness studies on recruitment strategies. Recruitment strategy selection will also need to respond to the needs and perspectives of participants, researchers, and clinicians [26,27].

We present the landscape of high-level EHR-driven recruitment strategies and an associated decision guide when evaluating differentiating factors. This guide will be more effective when used in consultation with a research informatics professional and incorporating key stakeholder perspectives [4]. We hope to educate researchers who may not be aware of the current capabilities and provide them a guide to better plan and implement a recruitment strategy that leverages the capabilities of the participating sites and meets the needs of the study protocol.
